# Successful Chemotherapy for Spontaneous Gastrosplenic Fistula in Diffuse Large B-Cell Lymphoma

**DOI:** 10.7759/cureus.92897

**Published:** 2025-09-22

**Authors:** Yuto Mimura, Ruiko Yamano, Yui Chikagawa, Takashi Kagaya, Kinya Ohata

**Affiliations:** 1 Department of Hematology, National Hospital Organization (NHO) Kanazawa Medical Center, Kanazawa, JPN; 2 Department of Gastroenterology, National Hospital Organization (NHO) Kanazawa Medical Center, Kanazawa, JPN

**Keywords:** chemotherapy (ct), diffuse large b cell lymphoma (dlbcl), gastrosplenic fistula, nonsurgical treatment, r-chop regimen

## Abstract

Gastrosplenic fistula (GSF) is a rare condition associated with gastric or splenic lymphomas. Surgical resection is the most commonly reported treatment, as has been documented in previous studies. We herein report a case of GSF in a 59-year-old man with diffuse large B-cell lymphoma (DLBCL). The patient was successfully treated with chemotherapy alone without the need for surgical resection. Given the patient's advanced stage and the absence of gastrointestinal bleeding or perforation, chemotherapy was selected as the first-line treatment to avoid the risks associated with surgery. The patient received the R-CHOP regimen (rituximab, cyclophosphamide, doxorubicin, vincristine, and prednisolone) along with high-dose methotrexate and intrathecal chemotherapy. No complications related to the fistula were observed during treatment, and the patient achieved complete metabolic remission.

## Introduction

Gastrosplenic fistula (GSF) is a rare and potentially fatal complication in lymphoma patients that is often associated with significant risks, such as gastrointestinal bleeding or perforation, and therefore may lead to severe morbidity if not promptly managed. It is most commonly associated with gastric or splenic involvement, particularly in cases of diffuse large B-cell lymphoma (DLBCL) [1-3]. Although uncommon, a systematic review of GSF has been published, indicating that surgical resection is the most frequently employed treatment approach [3].

We herein report a case of GSF in a patient with DLBCL that was successfully treated with chemotherapy alone, thereby avoiding surgical intervention and any of the morbidity associated with it.

## Case presentation

Initial presentation and medical history

A 59-year-old man presented with a two-week history of a fever. His medical history included stable mild ulcerative colitis for which he was taking mesalazine as his only regular medication. He reported no weight loss, upper abdominal pain, hematemesis, or melena.

Physical examination and laboratory findings

On a physical examination, he was febrile (39.4°C) and tachycardic, with otherwise stable vital signs. Splenomegaly was noted, with the spleen palpable 4 cm below the costal margins. There was no peripheral lymphadenopathy or sign of respiratory or cardiovascular disease. Laboratory tests showed normal hemoglobin (13.2 g/dL) and platelet count (314×10⁹/L), with mild leukopenia (white blood cell count: 3.4×10⁹/L, 87.1% neutrophils, 10.8% lymphocytes, 1.8% monocytes, and 0.3% eosinophils). A biochemical analysis revealed markedly elevated lactate dehydrogenase (1,800 U/L) and soluble interleukin-2 receptor (2,430 U/mL) levels, as well as elevated C-reactive protein (14.58 mg/dL). The renal function and electrolyte levels were within the normal limits. A summary of the laboratory results on presentation is shown in Table 1.

**Table 1:** Laboratory Results on Presentation With Reference Ranges H = high; L = low. Reference ranges may vary slightly by institution.

Parameter	Result	Unit	Reference Range
White blood cell count	3.4 L	×10⁹/L	4.5–9.0
Segmented neutrophils	87.1	%	10.0–90.0
Lymphocytes	10.8	%	5.0–60.0
Monocytes	1.8 L	%	5.0–10.0
Eosinophils	0.3	%	0.0–10.0
Hemoglobin	13.2	g/dL	13.0–17.1
Platelet count	314	×10⁹/L	150–350
Lactate dehydrogenase	1800 H	U/L	119–229
Blood urea nitrogen	12	mg/dL	8.0–22.0
Creatinine	0.49	mg/dL	0.60–1.00
Uric acid	2.8 L	mg/dL	3.6–7.0
Sodium	140	mmol/L	135–149
Potassium	4.2	mmol/L	3.5–4.9
Chloride	100	mmol/L	96–108
C-reactive protein	14.58 H	mg/dL	0.0-0.40
Soluble interleukin-2 receptor	2430 H	U/mL	157–474

Imaging studies

**Figure 1:**
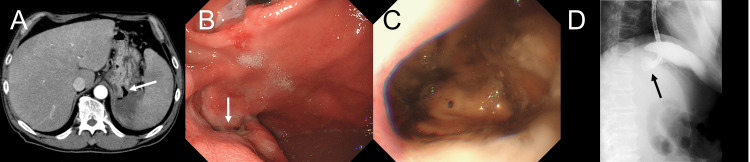
CT, EGD, and Gastric Fluoroscopy With a Gastrografin Injection Images. (A) Abdominal contrast-enhanced computed tomography (CT) shows the fistula (white arrow), which penetrates the dorsal wall of the gastric fundus and establishes a continuous connection with the gas-liquid cavity in the spleen. (B) Esophagogastroduodenoscopy (EGD) shows an opening of the fistula at the gastric fundus (white arrow). (C) A close-up EGD view showing the entire fistula opening. (D) Gastric fluoroscopy with a gastrografin injection shows the fistula between the stomach and spleen without any contrast leakage (black arrow).

Contrast-enhanced abdominal computed tomography revealed a bulky tumor measuring 6 cm in maximum diameter, forming a fistulous connection between the stomach and the spleen (Figure 1A). The tumor presented as a single confluent mass extending contiguously from the posterior wall of the gastric fundus to the pancreatic tail and splenic hilum, with suspected involvement of the left adrenal gland and splenic flexure. Multiple enlarged lymph nodes were identified along the lesser curvature of the stomach as well as in the peripancreatic and para-aortic regions. In addition, multiple hypovascular space-occupying lesions were observed in the liver. Esophagogastroduodenoscopy (EGD) revealed a large ulcerative lesion on the posterior wall of the gastric fundus (Figures 1B, 1C), and a biopsy was performed. Gastric fluoroscopy with a gastrografin injection confirmed the presence of a fistula between the stomach and spleen, without any evidence of contrast leakage into the peritoneal cavity (Figure 1D).

Diagnosis and treatment decision

A histopathological examination revealed a diffuse proliferation of abnormally large cells with nuclear swelling and chromatin condensation. Immunohistochemical staining showed that the tumor cells were positive for CD20, CD79a, and MUM1, and negative for CD3, CD5, CD10, BCL2, and BCL6. Epstein-Barr virus RNA was not detected by in situ hybridization. The Ki-67 labeling index was 60%-70%. Based on these findings, the tumor was diagnosed as DLBCL, a non-germinal center B-cell subtype. Staging was classified as stage IV disease (Lugano classification), with a high-risk prognosis based on the International Prognostic Index (IPI), National Comprehensive Cancer Network-IPI (NCCN-IPI), and Central Nervous System IPI (CNS-IPI). Although surgical resection is often reported as a treatment option for GSF, particularly in cases with common surgical indications such as either gastrointestinal bleeding or perforation, our patient did not present with these issues and therefore was clinically stable. This patient had stage IV disease with a bulky mass and suspected involvement of the adjacent organs. The presence of an active fistula and elevated inflammatory markers suggests a high risk of postoperative complications. Given the chemosensitive nature of DLBCL and the potential morbidity associated with extensive surgical resection, a multidisciplinary team including gastroenterologists, surgeons, radiologists, and hematologists determined that systemic chemotherapy would be the most appropriate initial treatment.

Treatment course and clinical outcome

**Figure 2:**
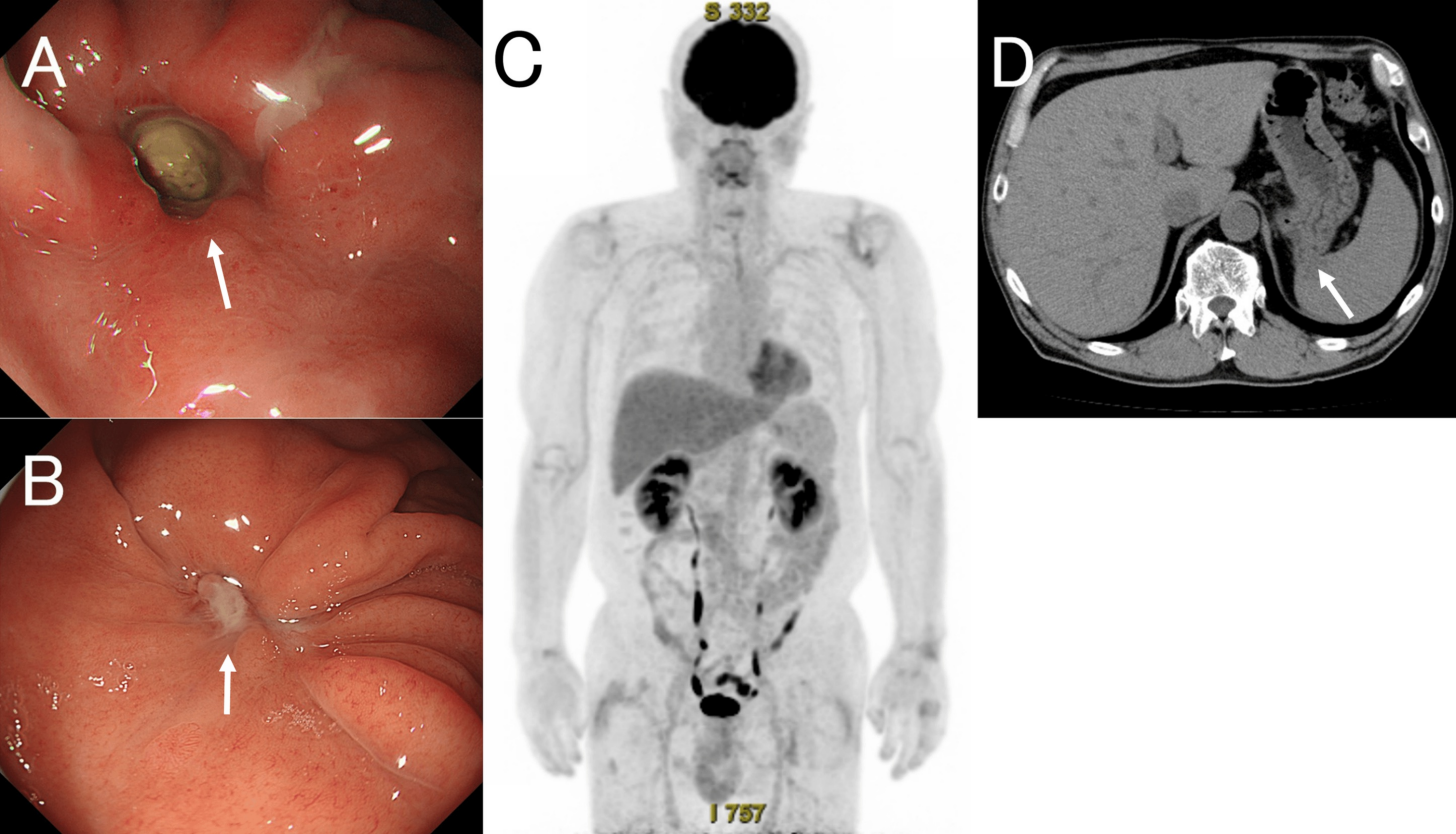
EGD and PET‐CT Images. (A) Esophagogastroduodenoscopy (EGD) after two cycles of chemotherapy shows the persistent fistula opening (white arrow). (B) EGD, performed six months after the initiation of chemotherapy, shows closure of the fistula (white arrow). (C, D) Positron emission tomography-computed tomography (PET-CT), performed seven months after the initiation of chemotherapy, shows no abnormal fludeoxyglucose F18 (FDG) uptake, consistent with complete metabolic remission. However, anatomical continuity between the stomach and spleen remains on CT (white arrow).

Chemotherapy was initiated with the CHOP regimen (cyclophosphamide 750 mg/m², doxorubicin 50 mg/m², vincristine 1.4 mg/m² on day 1, and prednisolone 100 mg/day on days 1-5). This led to a rapid improvement in symptoms and overall clinical status. No tumor lysis syndrome or disseminated intravascular coagulation was observed. Following the initial CHOP cycle, the patient received two cycles of R-CHOP (rituximab 375 mg/m² on the day before the CHOP regimen). This was followed by two cycles of rituximab (375 mg/m² on day one) with high-dose methotrexate (3.5 g/m² on day two), and then three additional cycles of R-CHOP. Intrathecal chemotherapy (methotrexate 15 mg, cytarabine 40 mg, and prednisolone 10 mg) was administered four times during the R-CHOP phase. This was not considered to be a major deviation from the standard regimen. Owing to the high-risk classification according to the CNS-IPI, both intrathecal chemotherapy and systemic methotrexate were administered for central nervous system prophylaxis, as recommended by the established clinical guidelines. After the initial CHOP cycle and one R-CHOP cycle, EGD demonstrated significant regression of GSF (Figure 2A), enabling the resumption of oral intake. Follow-up EGD after the completion of chemotherapy confirmed complete closure of the fistula (Figure 2B). Concurrent fluorodeoxyglucose-positron emission tomography-computed tomography (PET-CT) showed complete metabolic remission (Figures 2C, 2D). The patient remained in remission for two years after completing chemotherapy.

## Discussion

GSF is a rare complication that can occur in gastric or splenic lymphomas, particularly DLBCL [1-3]. A review from 1983 to 2016 indicated that the majority of GSF cases were DLBCL (85.2%), reflecting the predominance of DLBCL among primary gastric and splenic lymphomas [3]. Surgical resection is the first-line treatment for GSF [1-4]. In a review of 27 GSF cases associated with lymphoma, 88.9% of the patients underwent surgical resection, while only 7.4% were treated with chemotherapy alone [3]. However, in gastric DLBCL, chemotherapy (with or without radiotherapy) is commonly employed as the first-line treatment, with surgical resection typically reserved for complications such as bleeding or perforation [5].

Between February 2017 and June 2025, 12 cases of DLBCL that initially presented with GSF were reported. These cases are summarized in Table 2 [6-15]. Two cases were excluded due to the lack of active treatment: one patient died before therapy was initiated, and the other was transitioned to palliative care, rendering outcome comparisons unfeasible. Among the 10 evaluable cases, 60% underwent surgical resection, and 20% received chemotherapy alone. Notably, patients without signs of hematemesis or melena were more likely to be treated with chemotherapy than with surgery. Although surgical resection has been the preferred approach for GSF in DLBCL, largely because of concerns about gastrointestinal bleeding or perforation, it is not an established standard due to the rarity of this condition. Compared to previously reported cases (Table 2) [6-15], our patient did not present with either gastrointestinal bleeding or perforation, which are typically considered to be the main surgical indications. Considering the risk of complications and the chemosensitive nature of DLBCL, the patient’s overall clinical stability and the absence of any acute complications enabled us to initiate chemotherapy. Our case demonstrates that chemotherapy alone can be a viable and effective option in select patients without such complications. However, chemotherapy is not without risks. In particular, rapid tumor shrinkage may either worsen or enlarge the fistula, thus potentially leading to gastrointestinal bleeding or perforation. Other risks include tumor lysis syndrome and infections. Therefore, careful patient selection and close clinical monitoring are essential for making treatment decisions. Future prospective studies are warranted to determine the optimal management strategy for GSF in DLBCL.

**Table 2:** Summary of 11 Cases of DLBCL Presenting With GSF, Including 10 Published Between February 2017 and June 2025 and the Present Case NA: not available

Reference	Age/Sex	Maximum diameter	Presentation	Treatment	Complication	Outcome
[6]	73/male	NA	Weight loss and left upper quadrant pain followed by upper abdominal pain	chemotherapy	None	NA
[7]	63/male	13.1 cm	Melena and fever	chemotherapy and radiation	gastric bleeding	Disease-free after treatment for 4 years
[8]	63/female	NA	Dark stools, coffee-ground emesis, anorexia, nausea, mild epigastric pain and shortness of breath	surgical therapy	NA	NA
[9]	76/female	10 cm	Fever, weight loss, left upper quadrant pain and melena	surgical therapy	None	Disease-free after treatment for 5 years
[10]	76/male	10.9 cm	Malaise, weight loss and lower left chest pain	NA	NA	NA
[11]	60/male	NA	Asymptomatic	surgical therapy and chemotherapy	None	Disease-free after surgical therapy for 28 months
[12]	NA	18.8 cm	Hematemesis, orthostatic syncope, night sweats, fever, fatigue, intermittent abdominal pain and weight loss	surgical therapy	NA	NA
[13]	52/male	NA	Malaise, weakness, weight loss, occasional night sweats, and foul-smelling dyspepsia	chemotherapy	None	Disease-free after treatment for 14 months
[14]	59/male	7 cm	Intermittent left upper and lower quadrant abdominal pain, dark urine, fatigue and nausea	splenic embolization, surgical therapy and chemotherapy	NA	Remission but died of complications after 3 years
[15]	72/male	17 cm	Left upper quadrant abdominal pain, weight loss, early satiety, poor appetite and fatigue	surgical therapy and chemotherapy	None	Remission 3 months after treatment initiation
Present Case	59/male	6 cm	Fever	chemotherapy	None	Disease-free after treatment for 2 years

## Conclusions

Although surgical resection has traditionally been preferred for GSF in DLBCL, primarily because of concerns about bleeding or perforation, our case suggests that chemotherapy alone may be a feasible and effective treatment option in carefully selected patients without such complications. Careful patient selection and close clinical monitoring are essential for successful non-surgical management. Further prospective studies are warranted to establish an optimal treatment strategy for this rare but serious condition.
